# 2517 Learning to LEAD: Leadership emerging in academic departments

**DOI:** 10.1017/cts.2018.216

**Published:** 2018-11-21

**Authors:** Janice L. Gabrilove, Cara D. Ventura, Layla Fattah, Elizabeth Howell, Michele Fredericks, Lisa Bloom, Byron Cryer, Helen Yin

**Affiliations:** Icahn School of Medicine at Mount Sinai

## Abstract

OBJECTIVES/SPECIFIC AIMS: Leadership is an essential and recognized team science competency. Modeled after the successful LEAD (Leadership in Emerging Academic Departments) program at University of Texas Southwestern (UTSW), ConduITS LEAD Program is designed to: (1) provide personal and professional development opportunities for participants; (2) promote organizational change through applied leadership skills; (3) provide a platform for integrating multiple disciplines and fostering interprofessional relationships among investigators and clinicians. METHODS/STUDY POPULATION: The 1-year structured LEAD program curriculum includes monthly interactive seminars covering: personal and situational leadership; unconscious bias; communication and influence; navigating personal conflict; negotiation and networking; selecting and managing the right team; teamwork; financing the academic mission, budgets and business plan development; strategic planning and vision; presentation skills. To foster the development of leadership skills participants engage in Hogan Assessments, individual and peer mentoring from an executive coach and self-directed learning activities and assignments. Completion of an individual Capstone leadership project empowers learners to enact practice change through the implementation of leadership concepts in practice. RESULTS/ANTICIPATED RESULTS: In collaboration with the Office of Academic Enrichment & Development (OADE), the first competitive RFA was issued in November of 2016. In total, 63 applications were received including: gender: 29 M: 34 F; URM: 10; Degrees: M.D. (40); Ph.D. (11); M.D./Ph.D. (6); M.D./M.P.H. (3); M.D./M.S.C.R. (2); PharmD (1); Departments: 19; Institutes/Centers: 12; MSHS: 3 sites. Through a competitive and rigorous application process, 24 junior faculty with evidence of leadership potential and trajectory were chosen to participate. The current cohort of LEAD participants joined in February 2017, and will complete the program in January 2018. Using qualitative and quantitative survey methodology, participants will be evaluated for self-reported change to attitudes, belief, skills and development of new relationships and collaborations. Submitted Capstone projects were mainly focused on implementing situational and personal leadership concepts to practice, with one additionally focused on the use of behavioral interviewing techniques to optimize team building and teamwork. At the time of abstract submission 30% of the cohort has implemented their Capstone project in practice. Participants will be followed-up in 6 months’ time to evaluate the impact of the LEAD program on their practice. Following a second RFA, 24/52 candidates have been selected as our next cohort, and will start in February 2018. DISCUSSION/SIGNIFICANCE OF IMPACT: Leadership is known to be a core component of team science, and the ability to implement leadership into practice may advance personal and professional change. This program addresses the need to empower Junior Faculty to engage in leadership in practice. In addition, this program is able to provide added value to extend the reach of the OADE, promote new individual collaborations and facilitate additional leadership training efforts at our Institution. Future collaborative studies will focus on common outcomes as well as institutional differences between these 2 CTSA institutions.

